# Mapping the Centimeter-Scale Spatial Variability of PAHs and Microbial Populations in the Rhizosphere of Two Plants

**DOI:** 10.1371/journal.pone.0142851

**Published:** 2015-11-23

**Authors:** Amélia Bourceret, Corinne Leyval, Chantal de Fouquet, Aurélie Cébron

**Affiliations:** 1 CNRS, LIEC UMR7360, Faculté des Sciences et Technologies, Bd des Aiguillettes, BP70239, 54506 Vandoeuvre-lès-Nancy, France; 2 Université de Lorraine, LIEC UMR7360, Faculté des Sciences et Technologies, Bd des Aiguillettes, BP 70239, 54506 Vandoeuvre-lès-Nancy, France; 3 MINES ParisTech, Centre de Géosciences Géostatistique, Ecole Nationale Supérieure des Mines de Paris, 35 Rue Saint-Honoré, 77305 Fontainebleau, France; Leibniz-Institute of Vegetable and Ornamental Crops, GERMANY

## Abstract

Rhizoremediation uses root development and exudation to favor microbial activity. Thus it can enhance polycyclic aromatic hydrocarbon (PAH) biodegradation in contaminated soils. Spatial heterogeneity of rhizosphere processes, mainly linked to the root development stage and to the plant species, could explain the contrasted rhizoremediation efficiency levels reported in the literature. Aim of the present study was to test if spatial variability in the whole plant rhizosphere, explored at the centimetre-scale, would influence the abundance of microorganisms (bacteria and fungi), and the abundance and activity of PAH-degrading bacteria, leading to spatial variability in PAH concentrations. Two contrasted rhizospheres were compared after 37 days of alfalfa or ryegrass growth in independent rhizotron devices. Almost all spiked PAHs were degraded, and the density of the PAH-degrading bacterial populations increased in both rhizospheres during the incubation period. Mapping of multiparametric data through geostatistical estimation (kriging) revealed that although root biomass was spatially structured, PAH distribution was not. However a greater variability of the PAH content was observed in the rhizosphere of alfalfa. Yet, in the ryegrass-planted rhizotron, the Gram-positive PAH-degraders followed a reverse depth gradient to root biomass, but were positively correlated to the soil pH and carbohydrate concentrations. The two rhizospheres structured the microbial community differently: a fungus-to-bacterium depth gradient similar to the root biomass gradient only formed in the alfalfa rhizotron.

## Introduction

Polycyclic aromatic hydrocarbons (PAHs) are soil persistent organic pollutants whose main sources are the anthropogenic activities linked to the coal industry. Soil PAH contamination is generally heterogeneously distributed at different scales. While being spread at the kilometer scale [1], PAHs can display a heterogeneous distribution leading to pollution hotspots at smaller scales due to the presence of tar balls ([2,3]), to soil parameters, i.e. texture [4], organic matter content [5], mineral composition [6], and to PAH compound physico-chemical properties, i.e. molecular weight, solubility [7], molecular structure [8], governing adsorption, desorption and biodegradation mechanisms. Biological parameters such as plant growth and root development, and microbial community abundance and diversity, can also modify PAH distribution in the soil due to hotspots of activity in the rhizosphere.

Plants can contribute to the remediation of PAH-polluted soils. During plant growth, roots release exudates mainly composed of sugars, organic acids and amino acids ([9,10,11]) that constitute carbon sources for microorganisms. Root exudates can enhance PAH bioavailability ([12,13,14]), increase and activate rhizospheric microbes, and help them to degrade pollutants ([15,16]) by selecting PAH-degraders ([17,18]). Thus several studies carried out in artificially ([19,20,12,21]) or historically [22] contaminated soils report that PAH degradation is higher in plant rhizosphere than in non-planted soil. However, other studies report that addition of ryegrass root exudates did not modify [17] or even reduced or inhibited [23] PAH degradation, as compared to unplanted soil. Spatial and temporal variability of rhizospheric phenomena depending on root age and root exudate composition could potentially explain these contrasting observations.

On the other hand, the PAH remediation potential varies according to plant species [24]. Soil root structuration, root exudate concentration and composition, humidity, and chemical modifications induced by roots, depend on plant species type and could contribute explaining the temporal and spatial variations of rhizospheric processes during plant development [25]. The diversity of the rhizosphere microbial community, potentially involved in pollutant biodegradation, is linked to root exudate composition, which in turn depends on plant species ([26,27]), root age, and soil characteristics [28]. Different plant species have been tested in PAH rhizoremediation assays ([29,15,30]). Leguminous and grass plants are often selected because they are able to grow on nutrient-poor soils. Grasses develop a large fibrous root system that allows them to colonize a large area and favors interaction between roots, microorganisms and pollutants [29]. The impact of plants on PAH dissipation seems to vary according to proximity to growing roots [31], with a mm- to the cm-scale spatial gradient of bacterial communities [32]. Similar PAH gradients were observed in the rhizosphere of different plant species in rhizoboxes [33] and in field study [34]. The heterogeneous diffusion of root exudates, as shown in alfalfa rhizosphere [35], could also explain the spatial heterogeneity in PAH dissipation, but was not shown in a polluted soil.

Although numerous microcosm experiments using spiked soils have been designed to study temporal and spatial variability of rhizospheric processes, most devices have so far focused on distance gradients from the root but did not study the structuration at the whole root system or rhizosphere scale. The spatial variability of rhizospheric processes along the entire root system, taking into account roots of different ages and plants belonging to different families, is not yet well understood. These aspects could be further investigated using new combinations of tools, including visualization and modelling of the measured processes at the centimeter scale corresponding to the whole plant rhizosphere.

Geostatistical tools make it possible to characterize and quantify spatial variability. They are commonly used to study PAH contamination at the regional or site scale. Variogram studies have been used to highlight short distances spatial variability of hydrocarbon concentrations in contaminated soils with studies performed at the meter to hectometer scale ([36,37,38]), and at the decimeter to centimeter scale ([39,40]). These studies showed large spatial variability of hydrocarbon concentrations at short distances, with a decimeter-range structure. Geostatistical tools are also used to describe and establish spatial links between PAH contamination and soil physical, chemical and biological characteristics. Positive relationships between PAH hotspots and organic carbon concentrations [41], fungal biomass [42], bacterial diversity and ring-hydroxylating dioxygenase gene abundance [43], was established at the meter scale in aged-polluted soils. At finer (millimeter to centimeter) scale in urban roadside soil, the spatial heterogeneity of PAH-mineralization potential [3] was highlighted, but the distribution of PAHs and PAH-degraders was not correlated [44]. In contrast, other studies have demonstrated covariation between PAH concentration and spatial pattern of microbial diversity ([45,46]).

Although few studies used geostatistical tools to link pollutants with microbial community in PAH-contaminated soils, to our knowledge none of them considered plant rhizosphere at the centimeter scale, mapping the whole root system and its impact on the fate of PAHs.

We hypothesized that contrasted results concerning PAH-biodegradation in the rhizosphere are linked to spatial heterogeneity of rhizosphere processes, depending on plant species and root age, and varying with depth into the soil. To test this hypothesis, we developed a rhizotron device to study the rhizosphere at the centimeter-scale in order to consider and sample the whole root system. The device was adapted to visualize the rhizosphere structure after c.a. 1 month of plant growth. Thus we sampled a large number of points and performed a geostatistical study to map rhizosphere processes as an output of the fate of PAHs. We used a historically aged PAH-contaminated soil in which we increased organic pollutant bioavailability by spiking it with a complex organic extract at the start of the experiment. We measured biological and physical parameters in the initial spiked soil and after plant incubation, but we only compared their spatial variability after plant incubation. We grew two plant species (alfalfa and ryegrass) in separate rhizotrons, and then we carried out a geostatistical study to model the spatial variability of PAH biodegradation, microbial (bacterial and fungal) abundance, PAH-degrading bacterial abundance and root growth in the whole rhizosphere at the centimetre-scale in order to accurately map these variables.

## Materials and Methods

### Soil preparation

The experiments were carried out on a PAH- and heavy metal-contaminated soil from an aged coking plant site (Neuves-Maisons (NM), Lorraine, France) previously described [47]. Coke production from coal is an industrial process performed to obtain a powerful combustible used in the reduction of iron ore to steel in blast furnaces. This process generates many sub-products during the coal combustion, including PAH containing tar. Soil texture, agronomic and chemical characteristics and PAH concentrations are presented in Table 1. The soil was air-dried and sieved at 2 mm before use. The soil contained 1224 mg of Σ16 PAHs and 40 mg of Σ15 cyclodextrin-extractable PAHs per kg of dw soil. PAH content was artificially increased by spiking the soil with a complex organic pollutant extract [48]. The extract was prepared by mixing chloroform (2.470 l) with NM soil (2.250 kg). After 1h, the chloroform organic pollutant extract (0.779 l) was collected and spread onto 0.450 kg of a new NM soil aliquot. This spiked soil was air-dried for 2 days with frequent mixing under a fume hood to remove residual chloroform. The spiked soil was mixed to 4.050 kg of NM soil (non-exposed to chloroform) in a one-tenth ratio and separated in two batches to fill both rhizotrons. Two rhizotron devices were filled with spiked soil (1524 mg of Σ16 PAHs per kg of dw soil). Fifteen aliquots from both batches of spiked soil (30 T_0_ sub-samples) were stored at -80°C for further analyses of T_0_.

**Table 1:** Soil characteristics and PAH contamination. Granulometry, agronomic and chemical properties were measured at the LAS-INRA laboratory (Arras, France). Different letters indicate significant differences (Student t-test, p<0.05) in total and available PAH concentrations before and after spiking.

	Granulometry (g.kg^-1^)					Agronomic characteristics				Chemical characteristics					Σ16 PAHs (US-EPA) (mg.kg^-1^)			Σ15 bioavailable PAHs (mg.kg^-1^)	
Parameters	Clay (< 2 μm)	Fine silt (2/20 μm)	Coarse silt (20/50 μm)	Fine sand (50/200 μm)	Coarse sand (200/2000 μm)	N total (g.kg^-1^)	C/N	TOC (g.kg^-1^)	Organic matter (g.kg^-1^)		pH	CaCO_3_ (g.kg^-1^)	Available P_2_O_5_ (Olsen) (g.kg^-1^)	CEC (cmol+.kg^-1^)		Before spiking	After spiking	Before spiking	After spiking
NM soil	108	114	78	89	593	2.34	24.9	58.2	101		7.49	17	0.059	9.83		1224 ± 184 ^b^	1524 ± 174 ^a^	40 ± 13 ^b^	61 ± 15 ^a^

### Rhizotron device and sampling

**Fig 1:**
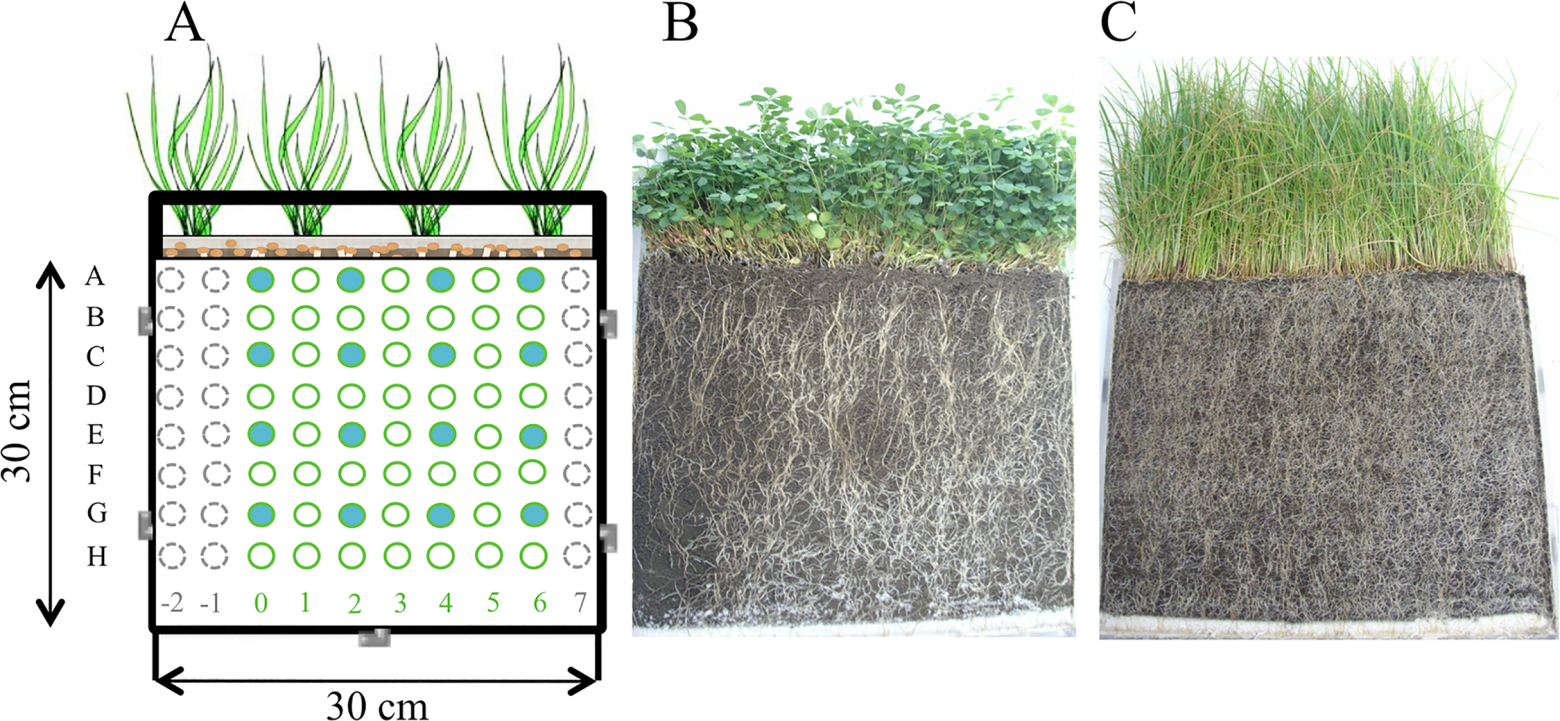
Illustration of the rhizotron device. (A) Sampling map showing the 56 samples (in green). The 8 depths are labeled from A to H and the 7 replicates are labeled from 0 to 6. Twenty-four supplemental samples (in gray) were used for soil humidity measurements. On sixteen samples per device (in blue) further analyses of pH, bioavailable PAHs, carbohydrates and dissolved organic carbon (DOC) were performed. Pictures of alfalfa (B) and ryegrass (C) rhizotron devices after 37 days.

Rhizotron devices were poly-methyl methacrylate boxes (30 cm high x 30 cm long x 2.5 cm wide) with a removable front face, and holes of 0.4 cm diameter at the bottom to avoid water stagnation. Bolting cloth (5 μl) was placed at the bottom and at the front face of the rhizotron to protect the root system and prevent it from adhering to the wall of the device. Two rhizotrons were filled with spiked soil and adjusted to 80% of the soil water holding capacity (WHC) (corresponding to 203 ml per kg of dw soil). Two plant species were grown in independent rhizotrons by seeding 4 g of alfalfa (*Medicago sativa* var. Europe; named Alf in the study) or ryegrass (*Lollium multiflorum* var. Podium; named Rye in the study) seeds (corresponding to 22.3 ± 2.0 seeds per cm^2^). After two days of seed germination, the devices were placed in a plant growth chamber with controlled conditions (22°C /18°C day/night, 80% relative humidity, c.a. 250 μmol photons m^-2^ s^-1^, 16 hours photoperiod) and were watered on the top to maintain soil humidity at 80% of the WHC by weighing the devices. After 37 days (T_37_), the front faces of the two rhizotrons were opened to allow sampling using a syringe whose bottom had been cut off (1.6 cm diameter, 2.5 cm long). We took 56 core samples per rhizotron (8 depths, 7 samples per depth), using a squared 3-cm grid pattern (3 cm between the core centers). Therefore we took a total of 112 samples for the two rhizotrons. The rhizotron device and the sampling grid are presented in Fig 1. For each sample, roots were separated and rinsed 3 times with sterile water to remove the adhering rhizosphere soil. Roots were air-dried at 60°C and weighed to estimate dry root biomass. The rhizosphere soil was collected from the first rinsing water. After centrifugation the rinsing water was removed and the rhizosphere soil was dried over night at room temperature. The ratio between rhizosphere soil and bulk soil was calculated by weighing the two soil fractions. Then rhizosphere soil was mixed to the bulk soil to obtain homogeneous soil samples. One gram of soil from 16 samples (4 samples from depths of 2, 8, 14, and 20 cm) was processed for available PAH extraction as described below, and all the other samples were stored at -80°C for further analyses. pH, total organic carbon and sugar concentrations were measured from these 16 soil solutions in distilled water (1:5 w/v ratio), respectively using a pH-meter electrode (BioBlock scientific, pHM210 Radiometer analytical), a total organic carbon analyzer (TOC-V CSH, Shimadzu) and an ion-exchange chromatograph (ICS 5000, Dionex, Thermo Scientific) equipped with a CarboPac SA10 column (Dionex, Thermo Electron). Carbohydrate analyses were performed at the certified facility in Functional Ecology (PTEF OC O81) from UMR 1137 EEF and UR 1138 BEF in the INRA Nancy-Lorraine research center. At each depth, 3 other samples were collected to measure soil humidity by weighing the soil sample before and after drying at 60°C for two days.

### Analysis of total and available PAHs

Total PAHs were extracted from 0.250 g of 30 T_0_ and 112 T_37_ samples of lyophilized soil, using an Accelerated Solvent Extractor (ASE350 Dionex ®) with dichloromethane (130°C, 100 bars). Available PAHs were extracted with 20 ml of an aqueous solution of hydroxypropyl-β-cyclodextrin (50 mM) (Acros organics, New Jersey, USA) [49] from 1 g of 8 T_0_ (4 aliquots from both batches) and 32 T_37_ samples of fresh soil in Teflon™ FEP (fluoroethylene propylene) Oak Ridge Centrifuge tubes (Nalgene, USA). After stirring on a rotary shaker for 16 h at 24°C and 2 centrifugation steps, PAHs were extracted from the aqueous supernatant (c.a. 30 ml) by adding 24 ml of dichloromethane. Dichloromethane extracts from total and available PAH fractions were evaporated under nitrogen flow and diluted in 20 ml and 5 ml of acetonitrile, respectively. PAHs were quantified by injecting 10 μl of extract in an HPLC (High-Pressure Liquid Chromatography, Dionex Ultimate 3000) system, using a 100-mm long and 4.6-mm internal diameter separation column (SupelcoAscendis Express C10), with 2.6-mm granulometry. A UV detector (254 nm) was used to individually detect the 16 US-EPA PAHs [50] for total extraction, and a fluorescence detector was used to detect 15 PAHs (acenaphtylene excluded) at low concentrations in the bioavailable extract.

### DNA extraction

Total nucleic acids were extracted from 0.5 g of the 6 T_0_ and 112 T_37_ samples of soil using a FastDNA SPIN Kit for soil (MP Biomedicals, France), and resuspended in 100 μl of DES (Dnase-free water). DNA concentrations and purity (A_260_/A_280_ ratio) were measured using a spectrophotometer (UV1800, Shimadzu) equipped with a Tray cell adapter (Hellma).

### Real-time PCR quantification of fungal, bacterial and PAH-degrading bacterial communities

The abundance of fungi, bacteria, and PAH-degrading bacteria was evaluated by real-time PCR as previously described ([51,52]), using the primer sets Fung5F /FF390R [53], 968F /1401R [54] and PAH-RHD_α_GN F/R, PAH-RHD_α_ GP F/R [51], which target the fungal 18S rRNA gene, the bacterial 16S rRNA gene, and the PAH-ring hydroxylating dioxygenase (PAH-RHD_α_) gene from Gram-negative (GN) and Gram-positive (GP) bacteria, respectively. Amplification reactions (20 μa final volume) were performed using 10 μf of iQ SYBR green SuperMix (Bio-Rad), 0.8 μ (of each primer (10 μf), 0.4 μ of 3% BSA (bovine serum albumin) solution, 0.2 μo of DMSO (dimethyl sulfoxide), 0.08 μ, of T4 bacteriophage gene 32 product (MP Biomedicals, France) and 1 μ of ten-fold diluted DNA, corresponding to 3.1 to 7.8 ng. Quantification was performed using a CFX96 Real-Time PCR detection system (Bio-Rad). For a description of standard plasmid dilution series (10^8^ to 10^1^ copies μl^-1^) and temperature profiles, see Cébron et al. (2008) [51] and Thion et al. (2012) [52]. Data were expressed in percentages of fungi or PAH-degrading bacteria relative to 16S rRNA gene copies.

### Statistical analyses

Statistical analyses were performed using XLStat 2013 software (Addinsoft). Fisher tests were performed to compare variance of the soil PAH content at T_0_ and at T_37_, and percentages of fungi to bacteria and Gram-negative PAH-degrading bacteria, respectively at depths 2 or 5 cm depth, according to each plant. Student t-tests were performed to determine statistical differences in available PAH (n = 16 samples per rhizotron), PAH concentrations, root biomass and abundance of the microbial (bacterial, fungal and PAH-degrading bacterial) community (n = 56 samples per rhizotron) over time, and to test for a plant effect. One-way analysis of variance (ANOVA, with p<0.05) followed by a Newmans-Keuls multiple comparison test was performed for each rhizotron to detect the impact of depth (n = 7 samples per depth). After standardization (z-score) of all data and labelling of samples according to their sampling coordinates independently of the rhizotron, a principal component analysis (PCA) was carried out, and a correlation circle was drawn. Correlation between soil properties was studied by calculating Pearson’s correlation matrix (S1 Table). Correlation between the parameters and depth (or root biomass) was described using the correlation ratio η^2^ in addition to the Spearman’s correlation coefficient r (η^2^ is equal to r^2^ if the correlation is linear, and is greater than r^2^ in the other cases) because the relationship between measured parameters and depth (or root biomass) is not necessarily linear.

### Geostatistical analysis

Geostatistics was used to study and model the spatial distribution of parameters that can be spatially structured in the soil, to verify if neighboring samples were more similar to one another than remote samples, i.e. if the data presented some kind of spatial autocorrelation. Many interpolation algorithms are available and generally provide different results (S1 Fig). Among those, linear optimal mapping (kriging) is defined by two criteria (i) non-bias (the average estimation error is zero), and (ii) precision (quantified by the variance of the estimation error, that must remain as low as possible).

Mapping was performed in two steps according to Chilès & Delfiner (2012) [55]: (i) an exploratory step of statistical and variographic analyses to characterize spatial variability, and (ii) a step of spatial variability modeling and estimation by kriging for mapping. The spatial structure of the variables was characterized by a sample variogram, which represents half of the mean quadratic difference (semivariance) between the values at two points according to their spacing. The sample variogram in the direction $\vec{s}$ is defined (putting $\vec{h}=h\;\vec{s}$) by $\gamma^{*}\;(\vec{h})=\frac{1}{2.n(\vec{h})}\sum_{i=1}^{n(\vec{h})}{\left(z(x_{i}+\vec{h})-z(x_{i})\right)}^{2}$, where x represents the experimental data points, z(x) the variable and $n(\vec{h})$ the number of point pairs separated by the distance $\vec{h}$. The behavior of the variogram with distance synthesizes the spatial variability of the variable z. The semi mean quadratic difference is usually low at short distances, and increases with distance. It can reach a sill when samples are independent from each other at greater distances than the range. Variogram maps were drawn to verify if data distribution followed a directional trend and revealed anisotropy, and directional variograms were calculated. Variograms were calculated at 3-cm steps (the data grid lag). ISATIS geostatistical software was used (http://geovariances.com).

Estimating the variable z apart from the data points requires modeling the sample variogram by a variogram function, noted $\gamma (\vec{h})$. This function $\gamma (\vec{h})$ was used to calculate the optimal linear estimator, also called “kriging”. As some variables are non-additive, “punctual” estimation, i.e. on the same support as the data, was carried on. Spatial distribution of soil parameters (PAH concentration, root biomass, microbial abundance) was modeled by kriging from a grid of 56 samples per rhizotron. Concerning the percentage of Gram-positive PAH-degrading bacteria in ryegrass, a moving 17-cm radius circular neighborhood, about half of the rhizotron, was selected. For all the other variables, kriging was performed in unique neighborhoods. The results of punctual kriging were slightly smoothed to draw the maps.

## Results

### Edaphic parameters and microbial community after 37 days

After thirty-seven days of plant growth (T_37_), roots had abundantly developed over the whole sampling area in the two rhizotrons. As expected with two plants belonging to two different families, root structure was plant specific (Fig 1). Soil humidity level, mannitol, xylose and fructose contents were significantly (p<0.05) higher in the ryegrass (59.5 ± 2.6%, 3.07 ± 2.03, 0.17 ± 0.23, 1.63 ± 1.02 mg.kg^-1^dw soil) than in the alfalfa (43.8 ± 7.4%, 1.18 ± 1.67, 0.00 ± 0.01, 0.28 ± 0.63 mg.kg^-1^dw soil) rhizotron (Table 2). The mannitol and fructose contents were lower at shallow depth in the ryegrass rhizotron. The dissolved organic carbon (DOC) and inositol contents were significantly higher in the alfalfa (189.73 ± 64.65, 0.64 ± 0.52 mg.kg^-1^dw soil) than in the ryegrass (121.34 ± 36.62, 0.16 ± 0.07 mg.kg^-1^dw soil) rhizotron. The inositol content was significantly higher at shallow depth for alfalfa, and specifically higher at 14 cm depth for ryegrass. pH, sucrose and glucose contents presented significant vertical gradients with depth. Globally, the soil was more acidic at shallow depth in both rhizotrons, whereas sucrose and glucose contents were lower at shallow depth in the ryegrass rhizotron only.

**Table 2:** Soil characteristics and carbohydrate concentrations after 37 days of alfalfa (Alf) or ryegrass (Rye) growth, at four depths (2, 8, 14, and 20 cm from the surface of the rhizotron). Values are means and standard deviations of three and four replicates for humidity and other parameters, respectively. The effect of plant species was evaluated using Student t-tests (p<0.05). The effect of depth was tested separately for each plant species after 37 days using a one-way analysis of variance (ANOVA) followed by Newman-Keuls multiple comparison test and letters (a, b, and c) indicate significant differences (p<0.05).

Conditions			Soil characteristics			Sugar concentrations (mg.kg^-1^)					
Samples	Plant	Depth(cm)	Humidity(%)	pH	DOC(mg.kg^-1^)	Inositol	Mannitol	Sucrose	Glucose	Xylose	Fructose
Alf_T37-2	Alfalfa	2	39.16 ± 4.99	6.91 ± 0.22 ^**b**^	210.16 ± 120.05	1.19 ± 0.23 ^**a**^	0.00 ± 0.00	0.28 ± 0.37	0.29 ± 0.43	0.00 ± 0.00	0.09 ± 0.13
Alf_T37-8	Alfalfa	8	42.34 ± 6.66	7.25 ± 57 ^**a**^	145.85 ± 29.92	0.32 ± 0.15 ^**b**^	1.45 ± 1.72	0.59 ± 0.87	0.21 ± 0.25	0.00 ± 0.00	0.10 ± 0.13
Alf_T37-14	Alfalfa	14	44.67 ± 7.85	7.34 ± 0.02 ^**a**^	180.13 ± 25.14	0.23 ± 0.18 ^**b**^	0.65 ± 0.96	0.55 ± 0.71	0.00 ± 0.00	0.00 ± 0.00	0.02 ± 0.04
Alf_T37-20	Alfalfa	20	44.40 ± 9.57	7.38 ± 0.03 ^**a**^	222.78 ± 15.93	0.82 ± 0.65 ^**ab**^	2.60 ± 2.23	3.34 ± 4.23	0.90 ± 1.09	0.01 ± 0.02	0.89 ± 1.13
Rye_T37-2	Ryegrass	2	57.46 ± 1.24	7.04 ± 0.15 ^**c**^	115.70 ± 39.70	0.16 ± 0.04 ^**ab**^	0.39 ± 0.48 ^**b**^	0.21 ± 0.26 ^**b**^	0.00 ± 0.00 ^**c**^	0.00 ± 0.00	0.66 ± 0.93 ^**b**^
Rye_T37-8	Ryegrass	8	58.35 ± 3.22	7.23 ± 0.03 ^**b**^	113.19 ± 32.19	0.07 ± 0.07 ^**b**^	2.84 ± 2.16 ^**a**^	0.98 ± 0.36 ^**a**^	0.27 ± 0.33 ^**bc**^	0.34 ± 0.18	1.44 ± 0.63 ^**ab**^
Rye_T37-14	Ryegrass	14	57.77 ± 1.63	7.32 ± 0.03 ^**ab**^	123.06 ± 43.92	0.24 ± 0.05 ^**a**^	4.73 ± 0.42 ^**a**^	1.17 ± 0.34 ^**a**^	0.82 ± 0.24 ^**a**^	0.20 ± 0.28	2.48 ± 0.93 ^**a**^
Rye_T37-20	Ryegrass	20	61.64 ± 1.75	7.40 ± 0.03 ^**a**^	133.43 ± 42.84	0.16 ± 0.03 ^**ab**^	4.33 ± 0.41 ^**a**^	0.81 ± 0.27 ^**a**^	0.62 ± 0.41 ^**ab**^	0.15 ± 0.26	1.93 ± 0.85 ^**ab**^
*Student t-tests*	Plant effect		Rye > Alf	n.s.	Alf > Rye	Alf > Rye	Rye > Alf	n.s.	n.s.	Rye > Alf	Rye > Alf

#### PAH contamination

**Fig 2:**
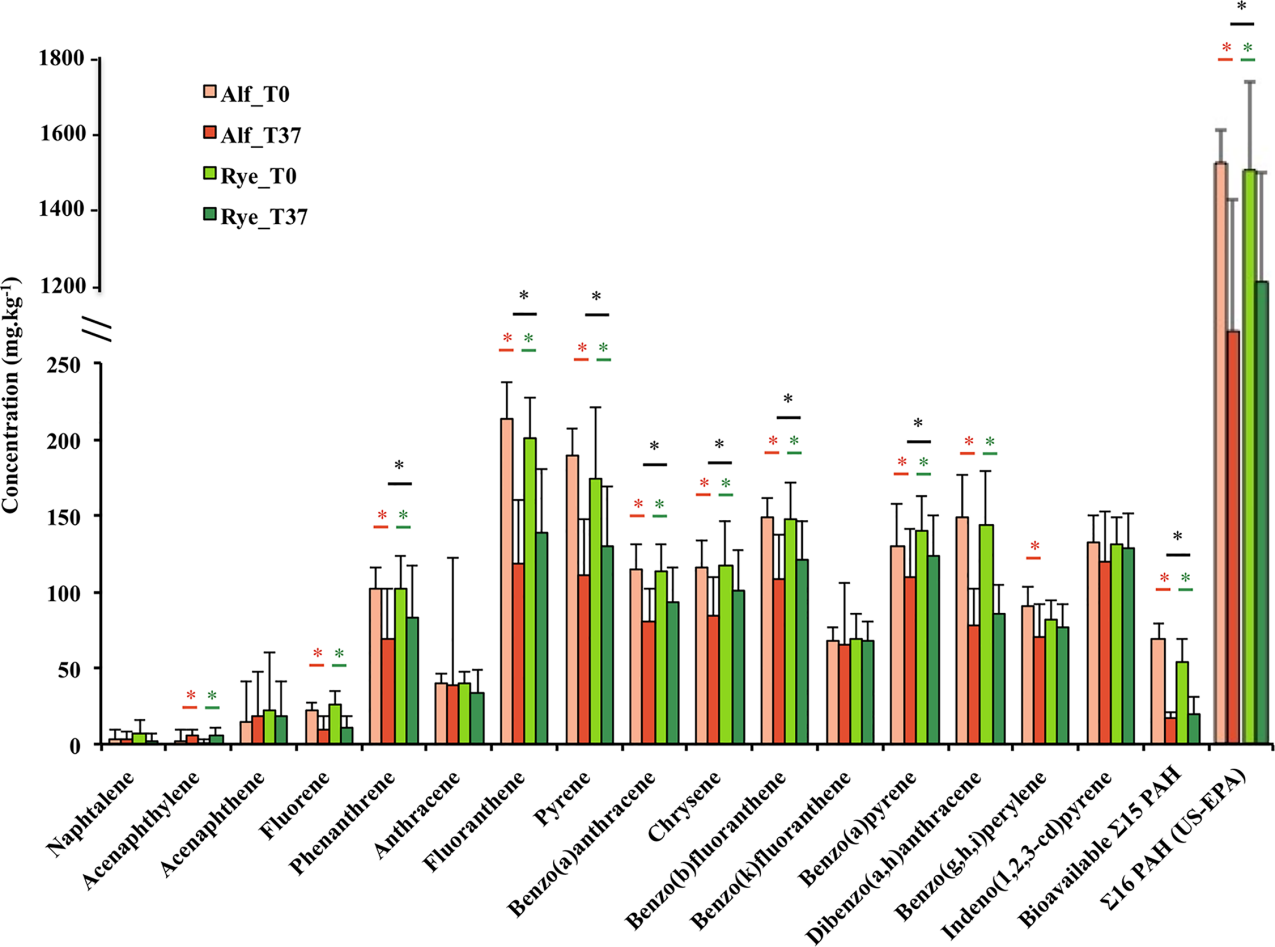
PAH contamination (sum of 16 US-EPA PAHs and sum of 15 available PAHs) over time (at T_0_ and at T_37_) and depending on the plant species (orange: alfalfa, Alf; green: ryegrass, Rye). Values are means and standard deviations of 15 and 56 replicates (PAHs), and 4 and 16 replicates (available PAHs) for the T_0_ and T_37_ conditions, respectively. The effects of time and plant species were evaluated separately using Student t-tests (p<0.05). Green and orange asterisks indicate significant changes in PAH concentrations between T_0_ and T_37_ in the ryegrass and alfalfa rhizotrons, respectively. Black asterisks indicate significant differences in PAH concentrations between the ryegrass and alfalfa rhizotrons at T_37_.

Spiking the soil with an organic pollutant extract significantly increased (p<0.05) the total PAH concentration in the soil from 1224 ± 184 mg.kg^-1^ dw soil before spiking to 1524 ± 174 mg.kg^-1^ dw soil at T_0_ just after spiking, i.e. a 20% increase in contaminants. The bioavailable PAH fraction was also increased by 34% (Table 1). The total PAH concentration decreased significantly (p<0.05) over time in both rhizotrons, down to 1090 ± 347 and 1220 ± 287 mg.kg^-1^dw soil in the alfalfa and ryegrass rhizotrons (T_37_), respectively, corresponding to 29% and 19% decreases relative to initial contamination of their respective T_0_ batches (Fig 2). At T_37_, total PAH concentration was significantly (p<0.05) lower in the alfalfa than in the ryegrass rhizotron. Most PAH concentrations decreased in both rhizotrons, independently of PAH molecular weight. However, at T_37_, the concentrations of seven compounds (phenanthrene, fluoranthene, pyrene, benzo(a)anthracene, chrysene, benzo(b)fluoranthene and benzo(a)pyrene) were significantly (p<0.05) lower with alfalfa than with ryegrass. Bioavailable PAH concentrations (th cyclodextrin-extracted PAHs) decreased significantly (p<0.05) over time in both rhizotrons, and reached 16 and 20 mg.kg^-1^dw soil at T_37,_ corresponding to 76% and 63% decreases for alfalfa and ryegrass, respectively, relatively to initial mean values of their respective T_0_ batches. With alfalfa only, the concentration in bioavailable PAHs was significantly (p<0.05) lower (16 ± 4 mg.kg^-1^dw soil) than in the original soil (40 ± 13 mg.kg^-1^dw soil) before spiking (Table 1).

#### Microbial community

**Fig 3:**
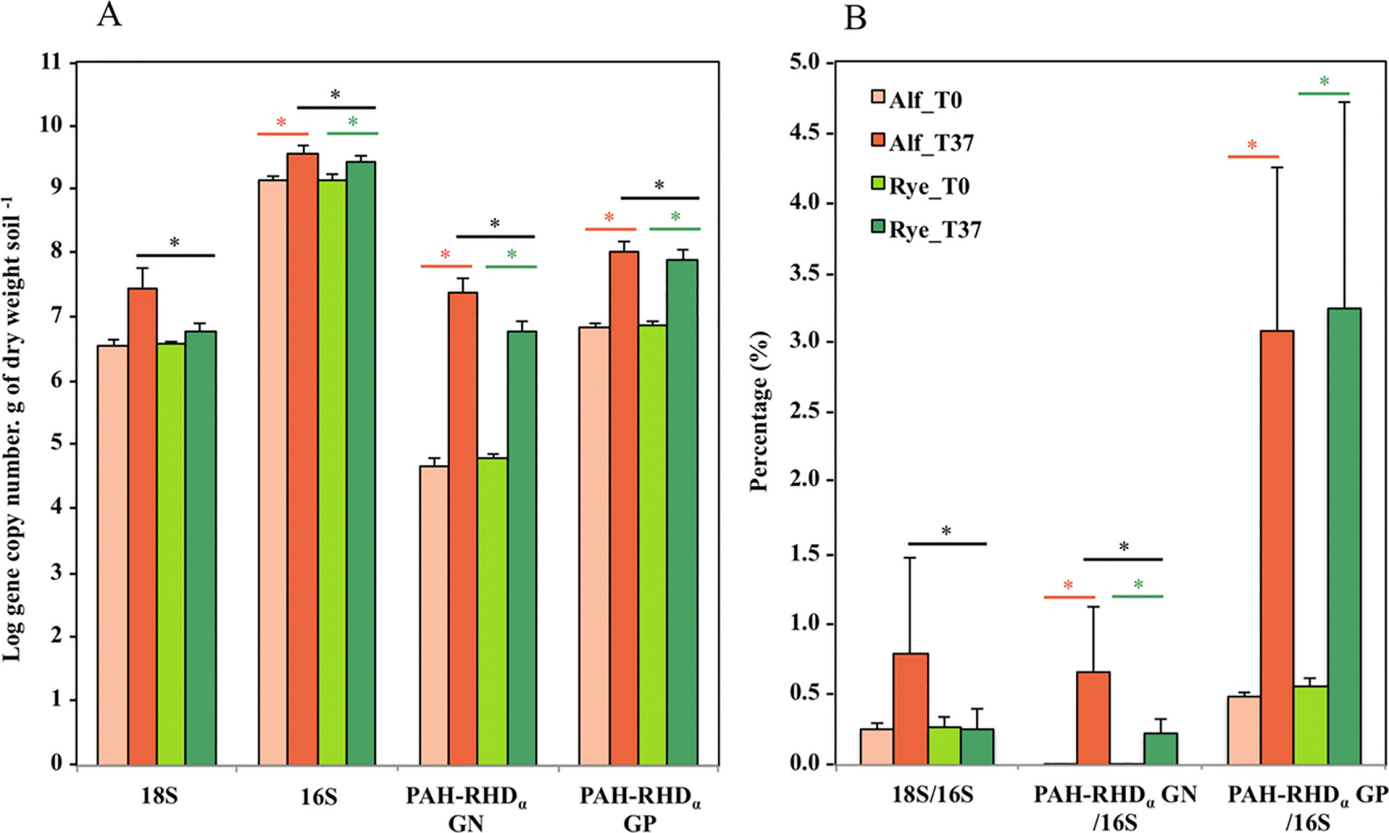
Abundance values (A) and percentages (B) (relative to 16S rDNA) of fungal, bacterial and PAH-degrading bacterial communities over time (at T_0_ and at T_37_) and depending on the plant species (orange: alfalfa, Alf; green: ryegrass, Rye). Values are means and standard deviations of 3 and 56 replicates for T_0_ and T_37_, respectively. The effects of time and plant species were evaluated separately using Student t-tests (p<0.05). Green and orange asterisks indicate significant changes in microbial community percentage or abundance between T_0_ and T_37_ in the ryegrass and alfalfa rhizotron, respectively. Black asterisks indicate significant changes in microbial community percentage or abundance between the two plants at T_37_.

At T_0_ and T_37_, bacterial abundance (mean value of 2.32 x 10^9^ ± 1.12 x 10^9^ 16S rRNA gene copies g dw soil^-1^) was two hundred times greater than fungal abundance (mean value of 1.08 x 10^7^ ± 1.26 x 10^7^ 18S rRNA gene copies g dw soil^-1^) (Fig 3). Bacterial and PAH-degrading bacterial abundance values increased significantly (p<0.05) over time and were significantly (p<0.05) higher at T_37_ for alfalfa than for ryegrass, and so were fungal abundance values. Globally, at T_37_ the PAH-degrading bacterial community was dominated by Gram-positive bacteria. The Gram-negative over Gram-positive ratios of bacterial PAH-degrading genes were 1:5 and 1:14 for alfalfa and ryegrass, respectively. The percentage of Gram-positive PAH-degrading bacteria increased 6-fold over time for both plants, while the percentage of Gram-negative PAH-degrading bacteria increased two-hundred fold only in the soil of the alfalfa rhizotron.

### Spatial variability of PAH, microbial community and rhizosphere parameters

#### Multivariate analysis

**Fig 4:**
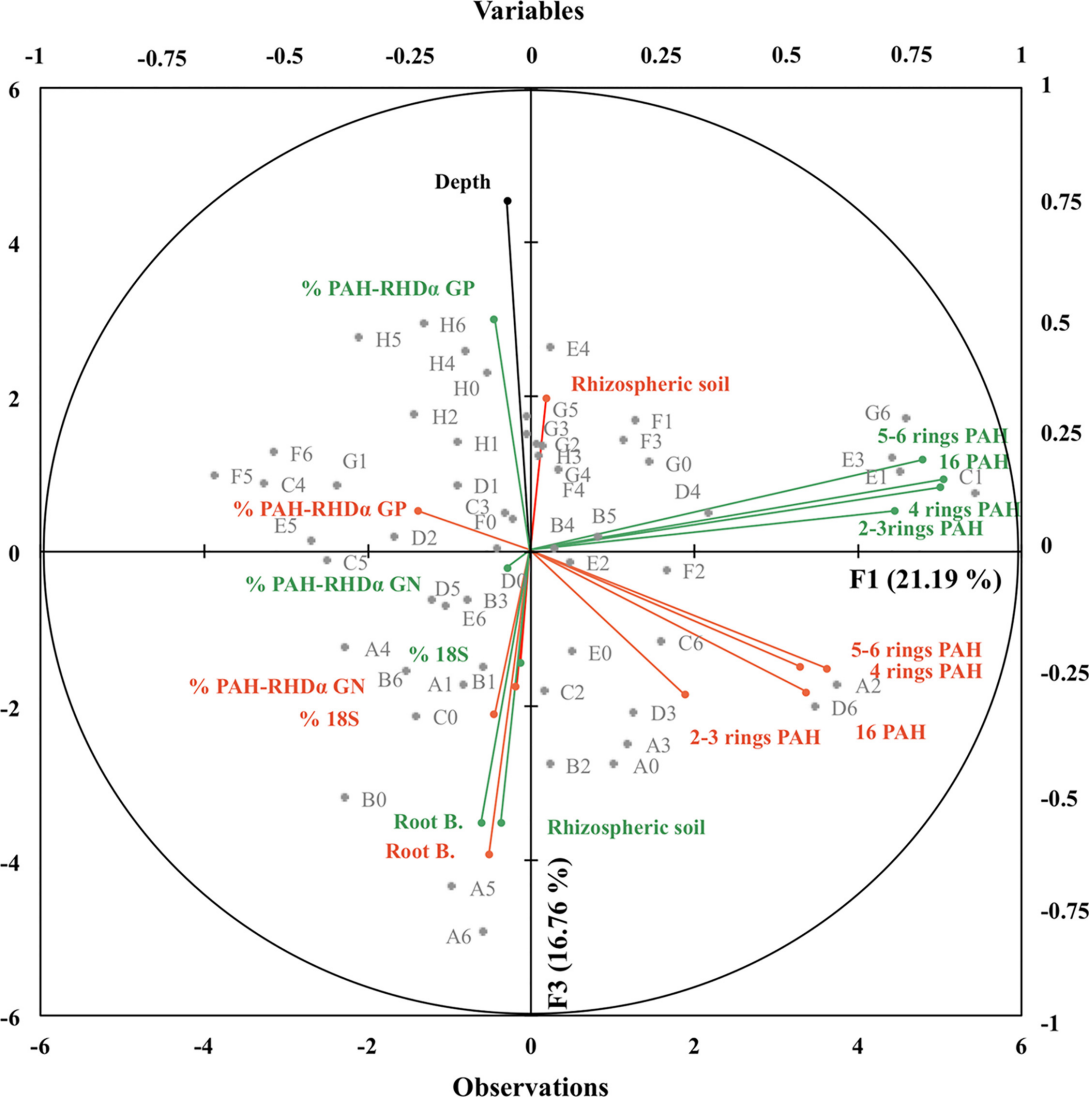
Principal component analysis (PCA) and correlation circle generated using depth, percentages (gene abundance relative to 16S rRNA gene abundance) of fungal (18S/16S) and PAH-degrading bacterial (% PAH-RHD_α_ GN and GP) communities, root biomass (Root B.), weight of rhizosphere soil, sum of 16 (US-EPA) PAH concentrations (16 PAHs), 2–3-ring PAHs (naphtalene, acenaphtylene, acenaphtene, fluorene, phenanthrene, anthracene), 4-ring PAHs (fluoranthene, pyrene, benzo(a)anthracene, chryzene), 5–6-ring PAHs (benzo(b)fluoranthene, benzo(k)fluoranthene, benzo(a)pyrene, dibenzo(a,h)anthracene, benzo(g,h,i)perylene, indeno(1,2,3-cd)pyrene) for the 56 samples (noted A to H for the 8 depths and 0 to 6 for the 7 replicates, as shown in Fig 1) from the alfalfa (orange) and ryegrass (green) rhizotrons.

Multivariate exploratory analysis was performed to highlight the parameters allowing discriminating samples. The two axes of the principal component analysis (PCA) taking into account depth, the percentage of fungi to bacteria, the percentage of Gram-negative and Gram-positive PAH-degrading bacteria, PAH concentration, root biomass, and weight of rhizosphere soil, explained 37.9% of total variability (Fig 4). The F3 axis (explaining 16.8% of the variability) was chosen instead of F2 axis (explaining 17.4% of the variability) because the F3 axis better separates the depth and plant parameters. On the correlation circle, biological (percentages of fungal and bacterial communities) and rhizosphere (weight of rhizosphere soil and root biomass) variables were separated from PAHs on the 1^st^ axis (F1). For each plant, PAHs (xis PAHs and 2–3, 4 and 5–6 cycles) were grouped together, indicating that all PAH concentrations evolved similarly over the 37 days, independently of their molecular weight. Separation of PAH concentration between the two plants on the 2^nd^ axis (F3) suggests that PAH concentrations were largely influenced by the plant species. The impact of depth on sample variability was also highlighted by the 2^nd^ axis (F3). Depth seemed negatively correlated with most biological variables similarly for both plants, particularly with the root biomass and proportion of rhizospheric soil. Negative and positive correlations of the percentage of fungi to bacteria in alfalfa, and the percentage of Gram-positive PAH-degrading bacteria in ryegrass were observed with depth, respectively.

#### Scatter plot analysis

**Fig 5:**
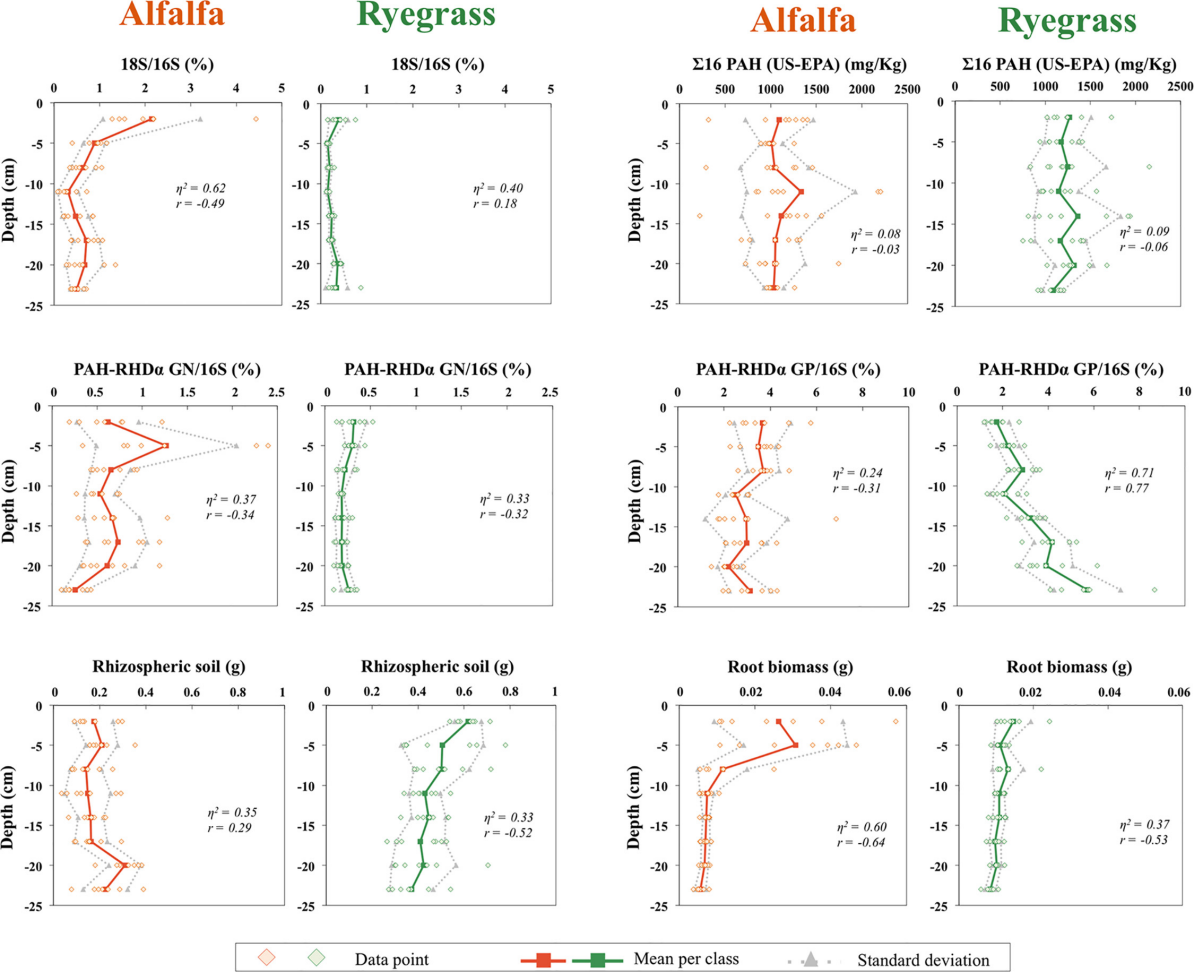
Relationship between percentages of fungal (18S/16S) and PAH-degrading bacterial communities (PAH-RHD_α_ GN and GP/16S), sum of 16 PAH concentrations, weight of rhizosphere soil, root biomass, and rhizotron depth for alfalfa (orange) and ryegrass (green). Correlation clouds show the data from the 56 soil samples (diamonds). Mean values (n = 7) per class (squares and full line) and class standard deviations (triangles and dotted lines) are represented. For each relationship, the Spearman’s correlation coefficient (r) and the non-linear correlation coefficient (η^2^) were calculated.

To better understand the impact of depth and root biomass on the other measured parameters, scatter plots were drawn and corresponding correlation coefficients given (Fig 5 and S2 Fig). At T_37_, the percentage of fungi to bacteria, and of Gram-positive PAH-degrading bacteria, as well as the root biomass appeared to be spatially structured by depth (Fig 5). The percentage of fungi to bacteria of the alfalfa rhizosphere (η^2^ = 0.62, r = -0.49) and the root biomass of the two rhizospheres (η^2^ = 0.60, r = -0.64; η^2^ = 0.37, r = -0.53) were negatively correlated with depth, whereas the percentage of Gram-positive PAH-degrading bacteria was positively (η^2^ = 0.71, r = 0.77) correlated with depth for ryegrass. More precisely, root biomass decreased regularly with depth in the ryegrass rhizosphere, whereas it was very much contrasted between the two shallow depths and the other depths in the alfalfa rhizosphere. In the alfalfa rhizotron, the percentage of fungi to bacteria and the percentage of Gram-negative PAH-degrading bacteria were greater in the first and second shallow depths, respectively. In the ryegrass rhizotron, these two parameters were significantly less dispersed at the same depths, and per-depth mean values were globally lower than in the alfalfa rhizotron. The percentages of Gram-positive PAH-degrading bacteria were differently distributed across space between the two plants although mean values were close (3.08 and 3.24% for alfalfa and ryegrass, respectively). Per-depth mean values tended to increase in the ryegrass rhizosphere, but not in the alfalfa rhizosphere. Thus, total PAH concentrations did not exhibit spatial co-variation with depth. We found no significant correlation between PAH concentrations, percentages of Gram-negative PAH-degrading bacteria and depth for either plant. Moreover, there was no significant co-variation between root biomass and microbial community and PAH concentration parameters, neither for alfalfa nor for ryegrass (S2 Fig).

#### Variographic analysis and mapping

**Fig 6:**
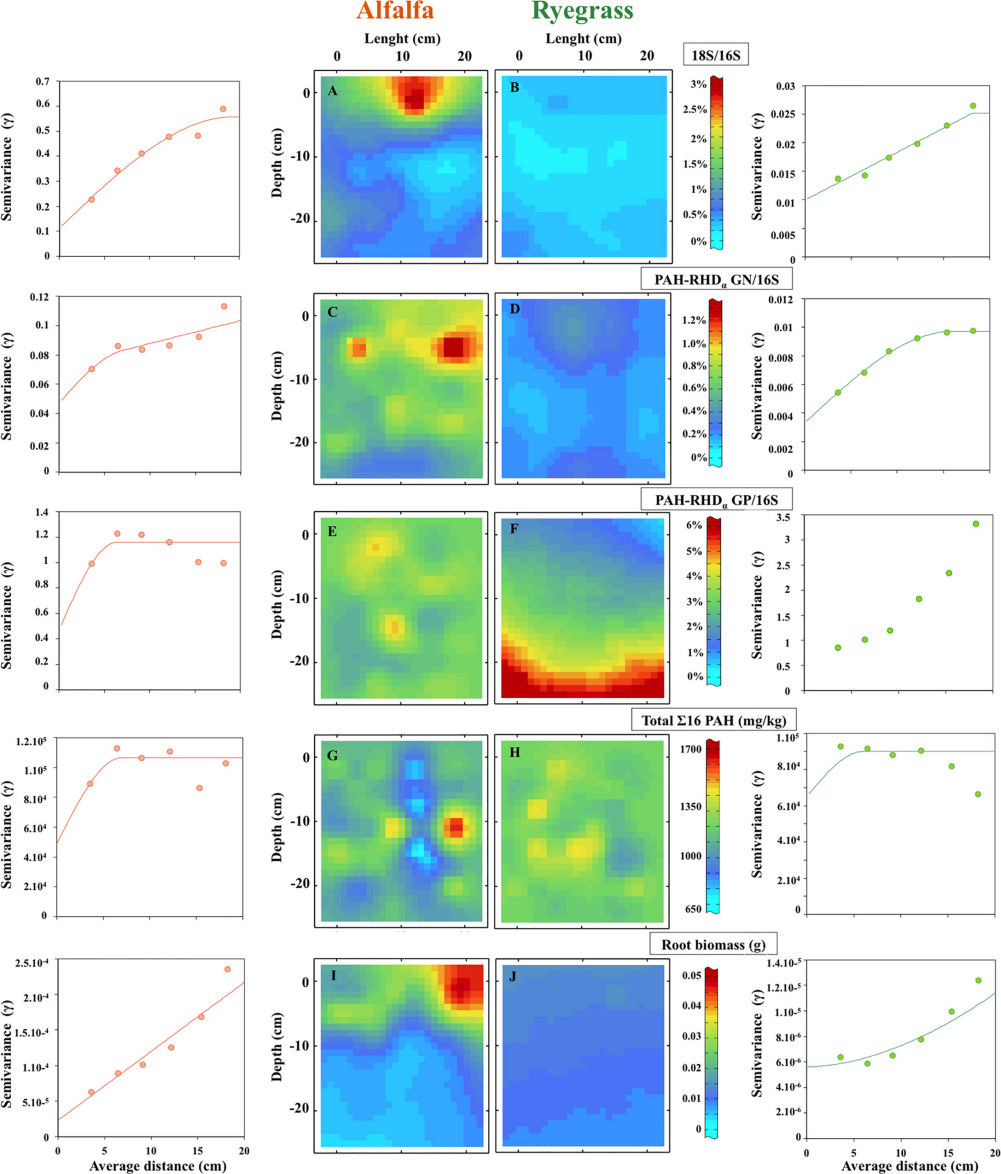
Semivariograms and kriging maps modeling the spatial distribution of 5 different parameters measured from the alfalfa (left) and ryegrass (right) rhizotrons. Data after 37 days for: (A and B) fungal communities (percentages of 18S rRNA genes relative to 16S rRNA genes), (C to F) PAH-degrading bacterial communities (percentages of PAH-RHD_αo_GN and PAH-RHD_α_ GP genes relative to 16S rRNA genes), (G and H) sum of 16 (US-EPA) PAHs, and (I and J) root biomass. Samples (circles) and fitted semivariograms (lines) are shown. Semivariance was plotted versus distances between samples, and for each parameter different models were used to fit the data (spherical model with nugget effect: alfalfa: sum of 16 PAHs, 18S/16S, PAH-RHD_α_ GP/16S; ryegrass: sum of 16 PAHs, PAH-RHD_α_ GN/16S; spherical model, order-1 G.C., with nugget effect: alfalfa: PAH-RHD_αN_GN/16S; order-1 G.C model with nugget effect: ryegrass: 18S/16S; power model with nugget effect: ryegrass: root biomass; linear model with nugget effect: alfalfa: root biomass. A sample variogram corresponding to PAH-RHD_αc_GP/16S data was fitted with the more general model of intrinsic random first order function. No model fitted the sample variogram for this variable. Contour maps (10 mm mesh) derived from values interpolated by punctual kriging of 56 samples. (N/A: non-available data).

Although directional variograms and variographic maps indicated anisotropy, isotropic modeling was chosen because anisotropy was low or impacted by a few large values, preventing map distortion by insufficiently robust modeling. Sample variograms of the percentage of Gram-positive PAH-degrading bacteria in alfalfa and of total PAHs for both plants were “flat”, with a large nugget effect (Fig 6). Automatic fitting was not used for the variogram of total PAH concentrations in the ryegrass rhizotron, and a short-range structured component was introduced to avoid a uniform map. Kriging estimation showed that data from close sampling point could be very different. Concerning root biomass in alfalfa and the percentage of fungi to bacteria in ryegrass, increasing variograms were automatically fitted with large range components: the non-stationarity of these parameters can be seen as spatial correlation to a large distance. Kriging estimation confirmed that root biomass was negatively correlated with depth in the alfalfa rhizosphere. Root biomass variogram indicates non-stationarity at a higher order. The percentage of Gram-negative PAH-degrading bacteria in the ryegrass rhizosphere presented parabolic variograms and fitting indicated a range of 15 cm. A larger range was observed for the percentage of fungi to bacteria, the percentage of Gram-negative PAH-degrading bacteria and root biomass in the alfalfa rhizosphere. Root biomass in ryegrass presented less stationary fitting, with a 1.8-exponent component. Kriging of data at T_37_ confirmed the negative depth gradient of the percentage of fungi to bacteria in the alfalfa rhizosphere. Moreover, mapping allowed us to visualize higher abundance of the fungal community in the alfalfa rhizosphere than in the ryegrass rhizosphere. Although the percentage of Gram-negative PAH-degrading bacteria and PAH dissipation were higher in the alfalfa than in the ryegrass rhizotron, no spatial structuring was observed with depth. The variogram fitting of the percentage of Gram-positive PAH-degrading bacteria in the ryegrass rhizosphere would have led to a parabola (exponent 2), which is an ineligible model. For this variable with such a degree of non-stationarity, fitting was performed within the more general model of an intrinsic random function of order one. The variogram was replaced by generalized, indirectly fitted covariance. Kriging of data at T_37_ confirmed the positive depth gradient of the percentage of Gram-positive PAH-degrading bacteria in the ryegrass rhizosphere.

## Discussion

### Spatial gradients in the rhizosphere

After 37 days, root development had induced a spatial heterogeneity in the biomass and age of roots depending on depth, for both ryegrass and alfalfa. Although root biomass was greater at shallow depth in both rhizotrons, the root gradient was more contrasted with alfalfa, probably due to plant species-dependent root system structures. Root growth induced both physical and chemical gradients across the rhizotrons that shaped the microbial community, but not much the PAH-degrading activity.

Soil humidity, a parameter linked to plant development that could influence microbial rhizospheric activity and potentially organic contaminant degradation, did not change significantly with rhizotron depth and was not sufficient to explain depth structuring. The pH was more acidic at shallow depths, where roots were more abundant, as previously shown [56]. The pH can have an influence on bacterial community composition [57]. Moreover, root exudate composition differed with depth, especially sugar concentrations. Although gradients for the different measured sugars were observed in both directions, we can’t exclude that root exudate leaching, due to rhizotron watering, can participate to depth gradients. Spatial gradient of exudate quality was probably linked to the developmental stage of the plant roots ([58,59]) that was different between the shallow and deep parts. Exploratory analysis to study the relationship between different parameters for a same sampling point showed that the percentage of fungi to bacteria was negatively correlated with depth in the alfalfa rhizotron, whereas the percentage of Gram-positive PAH-degrading bacteria was positively correlated with depth in the ryegrass rhizotron. Geostatistical modeling confirmed these trends and evidenced spatial variation of these two parameters with depth. As no strong correlation was shown between these parameters and root biomass, the measurement of root length and/or root surface could provide in future studies further details about the impact of root development. The plant-specific gradient induced by 37 days of root growth led to a significant shift in microbial community abundance and structure, as shown during the development of other plant species ([60,61,62]). Moreover, parameter variability in the rhizosphere, linked to root structure, can impact the microbial community by creating ecological microenvironments at a fine scale [63].

Despite the presence of a root biomass gradient, total PAH concentrations were not spatially auto-correlated and presented a nugget effect suggesting that, after 37 days of plant development (T_37_), roots did not impact noticeably PAH degradation and consequently PAH content at centimeter-scale. Yet, the PAH content variability was greater in alfalfa than in ryegrass rhizosphere, suggesting an impact depending on plant species. This unstructured distribution commonly occurs in PAH-contaminated soil from the meter [42] to the centimeter-scale [3]. Several studies suggest that this kind of heterogeneous contaminated environment impacts microbial communities distribution and activity. For instance, a geostatistical analysis at the centimeter scale showed that metal hotspots corresponded to low bacterial activity in a heavy-metal-contaminated soil [64]. On the contrary, characterization of spatial variability of microbial community structure in an aged creosote site showed that PAH hotspots corresponded to higher microbial biomass [45] and respiration rates [46]. Moreover these authors showed impact of PAH heterogeneity on bacterial community structure with increase of *Proteobacteria* and decrease of *Actinobacteria* abundances in PAH hotspots, suggesting modification in PAH-degrading communities. Other studies indicate that high concentration and hotspots of PAH and petroleum hydrocarbon contamination increased the abundance of PAH-degrading genes ([65,66,43]). In our study, centimeter-scale mapping revealed the presence of hotspots of PAH-degrading bacteria and of PAH content, but there was no obvious correlation between these two parameters. Similar results were obtained by Johnsen et al. (2014) [44], in a polluted roadside soil at the millimeter- to centimeter-scale. The lack of correlation could be explained by the thorough degradation of the spiked PAH fraction after 37 days, when mapping was performed.

### Comparison of PAH concentrations and microbial abundance in two contrasted rhizospheres

In aged contaminated soils, bioavailability is one of the most important parameters that limits PAH biodegradation [67]. Soil preparation by adding a soil organic extract increased total and bioavailability fractions by 20 and 34% respectively, while keeping the historical contaminants and the adapted microbial community [48]. The incubation of this contaminated soil in the presence of two different plants in a rhizotron device for 37 days under controlled conditions (humidity, temperature and light) showed a significant dissipation of PAHs as compared to the beginning of the assay. The decrease in PAH bioavailability within 37 days in both the alfalfa and ryegrass rhizotrons and the degradation rate suggested that PAH dissipation corresponded to all of the spiked and bioavailable fractions. After 37 days of incubation, PAH concentrations in the two rhizotrons were close but lower in alfalfa. Since no rhizotron without plant was included in our study, we cannot conclude that dissipation of PAHs was due to the plants. However, we observed that dissipation of total PAHs and of some high-molecular-weight compounds was significantly higher in the alfalfa than in the ryegrass rhizotron, based on analysis of the 56 samples per rhizotron. The relatively long duration of the experiment probably smoothed out the differences in PAH dissipation rates occurring during the first days or weeks, and maybe obliterated different responses of the two plant species. A supplementary kinetic approach could be interesting to set up the time-course of PAH dissipation and changes in bacterial communities. PAH dissipation can be explained by modifications in the microbial community structure and abundance, namely by an increase in the abundance of both Gram-positive and Gram-negative PAH-degraders during the 37 days of incubation, as compared to the beginning of the assay. This increase occurred in the two rhizotrons, whatever the plant species, as root exudates were shown to favor PAH-degrading bacteria in contaminated soils ([66,68,18,69]).

The devices were considered as homogeneous in PAH content at the centimeter scale at the beginning of the assay, even if hotspots due mainly to tar balls present from the aged-PAH contamination were observed (variability of PAH content values at T_0_ estimated on 30 independent sample measurement). Heterogeneity in PAH content due to spiking should appear at a finer scale (grain scale, mm) and was not observed at the cm-scale since spiking did not increase the variability of PAH measurement compared to the initial soil. Moreover, comparison of variances between T_0_ and T_37_ PAH values showed that alfalfa had an effect on PAH content variability, while ryegrass did not. Although we can not exclude that a similar phenomenon could have occurred without plant, a higher variability of PAH concentration at T_37_ than at T_0_ highlights an effect of alfalfa roots on PAH dissipation efficiency, as alfalfa accentuated the pollutant hot- and cold-spots. The difference between the two plants could be explained by specific and contrasted root structures. Alfalfa possesses a taproot system composed of a central root with emerging roots [70], while ryegrass is characterized by a highly branched and fibrous root system with many fine roots that impact differently the root-soil interface [71]. The differences between the two plants concerning dissolved organic carbon and sugar contents confirmed that root exudate composition is plant-specific as previously shown during a 6-week-long rhizotron experiment for free sugars and amino acids [72]. Root exudate quality provides a soluble and easily degradable carbon source to the soil, so it impacts microbial population abundance.

## Conclusion

Using two plants with contrasted root structures commonly used in phytoremediation trials, we showed that the whole spiked PAH fraction was dissipated after 37 days in a controlled rhizotron experiment. Such dissipation was associated with microbial community modification, namely the percentage of PAH-degrading bacteria increased over time. We used geostatistics to describe and quantify the soil spatial structure. Kriging mapping yielded a rational basis for comparing the two rhizospheres. It evidenced a root-dependent physical, chemical and biological spatial gradient. The percentages of Gram-positive PAH-degrading bacteria and of fungi were spatially auto-correlated in the alfalfa and ryegrass rhizotrons, respectively, and presented depth gradients. Although PAH concentrations exhibited a nugget effect, we recorded contrasting effects of plant species on PAH degradation that might be due to differences in microbial activity or diversity. These parameters should be further investigated to evaluate the impact of rhizosphere gradients on microbial diversity and activity over time in order to estimate their temporal variability.

## Supporting Information

S1 Fig Comparison of different mapping representations of the percentage of PAH-RHD_α_ GP relative to 16S rDNA variable for ryegrass. A: measured data point, B: data point estimated by nearest neighboring, C: kriging estimation, F: estimation by inverse distances, with 1/D^3^. N/A: non-available data.
(TIF)

S2 Fig Relationship between percentages of fungal communities (18S/16S), PAH-degrading bacterial communities (PAH-RHD_α_ GN and GP/16S), the sum of 16 PAH concentrations, weight of rhizosphere soil and root biomass for alfalfa (orange) and ryegrass (green). Correlation clouds show the data from the 56 soil samples (diamonds). Mean values (n = 7) per class (squares and full line) and class standard deviations (triangles and dotted line) are represented. For each relationship, the Spearman’s correlation coefficient (r) and the non-linear correlation coefficient (η^2^) were calculated.
(TIF)

S1 Table Pearson’s correlation matrix between soil properties. Values in bold correspond to significant correlation between variables (p<0.05).
(DOCX)

## References

[1] Villanneau E, Saby NA, Orton T, Jolivet C, Boulonne L, Caria G, et al First evidence of large-scale PAH trends in French soils. Environ Chem Lett. 2013;11: 99–104. 10.1007/s10311-013-0401-y

[2] De Fouquet C. From exploratory data analysis to geostatistical estimation: examples from the analysis of soil pollutants. Eur J Soil Sci. 2011;62: 454–466.

[3] Hybholt TK, Aamand J, Johnsen AR. Quantification of centimeter-scale spatial variation in PAH, glucose and benzoic acid mineralization and soil organic matter in road-side soil. Adapt For Ecosyst Air Pollut Clim Change. 2011;159: 1085–1091. 10.1016/j.envpol.2011.02.028 21396755

[4] Amellal N, Portal J-M, Vogel T, Berthelin J. Distribution and location of polycyclic aromatic hydrocarbons (PAHs) and PAH-degrading bacteria within polluted soil aggregates. Biodegradation. 2001;12: 49–57. 10.1023/A:1011909107858 11693295

[5] Cornelissen G, Gustafsson Ö, Bucheli TD, Jonker MTO, Koelmans AA, van Noort PCM. Extensive Sorption of Organic Compounds to Black Carbon, Coal, and Kerogen in Sediments and Soils: Mechanisms and Consequences for Distribution, Bioaccumulation, and Biodegradation. Environ Sci Technol. 2005;39: 6881–6895. 10.1021/es050191b 16201609

[6] Choudhary VR, Mantri K. Adsorption of Aromatic Hydrocarbons on Highly Siliceous MCM-41. Langmuir. 2000;16: 7031–7037. 10.1021/la991714u

[7] Styrishave B, Björklund E, Johnsen A, Halling-Sørensen B. The Spatial Heterogeneity of Polycyclic Aromatic Hydrocarbons in Soil Depends on Their Physico-chemical Properties. Water Air Soil Pollut. 2012;223: 969–977. 10.1007/s11270-011-0916-4

[8] Tunega D, Gerzabek MH, Haberhauer G, Totsche KU, Lischka H. Model study on sorption of polycyclic aromatic hydrocarbons to goethite. J Colloid Interface Sci. 2009;330: 244–249. 10.1016/j.jcis.2008.10.056 18996540

[9] Kumar R, Pandey S, Pandey A. Plant roots and carbon sequestration. Curr Sci. 2006;91: 885–890.

[10] Vančura V, Hovadik A. Root exudates of plants: II. Composition of root exudates of some vegetables. Plant Soil. 1965; 21–32.

[11] Chaudhry Q, Blom-Zandstra M, Gupta SK, Joner E. Utilising the Synergy between Plants and Rhizosphere Microorganisms to Enhance Breakdown of Organic Pollutants in the Environment (15 pp). Environ Sci Pollut Res. 2005;12: 34–48. 10.1065/espr2004.08.213 15768739

[12] Joner EJ, Corgié SC, Amellal N, Leyval C. Nutritional constraints to degradation of polycyclic aromatic hydrocarbons in a simulated rhizosphere. Soil Biol Biochem. 2002;34: 859–864. 10.1016/S0038-0717(02)00018-4

[13] Joner EJ, Leyval C. Rhizosphere gradients of polycyclic aromatic hydrocarbon (PAH) dissipation in two industrial soils and the impact of arbuscular mycorrhiza. Environ Sci Technol. 2003;37: 2371–2375. 12831019 10.1021/es020196y

[14] Sun B, Ling W, Wang Y. Can root exudate components influence the availability of pyrene in soil? J Soils Sediments. 2013;13: 1161–1169. 10.1007/s11368-013-0712-4

[15] Kuiper I, Lagendijk EL, Bloemberg GV, Lugtenberg BJ. Rhizoremediation: a beneficial plant-microbe interaction. Mol Plant Microbe Interact. 2004;17: 6–15. 14714863 10.1094/MPMI.2004.17.1.6

[16] Gerhardt KE, Huang X-D, Glick BR, Greenberg BM. Phytoremediation and rhizoremediation of organic soil contaminants: Potential and challenges. Plant Sci. 2009;176: 20–30. 10.1016/j.plantsci.2008.09.014

[17] Cébron A, Louvel B, Faure P, France‐Lanord C, Chen Y, Murrell JC, et al Root exudates modify bacterial diversity of phenanthrene degraders in PAH‐polluted soil but not phenanthrene degradation rates. Environ Microbiol. 2011;13: 722–736. 10.1111/j.1462-2920.2010.02376.x 21087382

[18] Storey S, Ashaari MM, McCabe G, Harty M, Dempsey R, Doyle O, et al Microbial community structure during fluoranthene degradation in the presence of plants. J Appl Microbiol. 2014;117: 74–84. 10.1111/jam.12518 24712542

[19] Liste H-H, Alexander M. Plant-promoted pyrene degradation in soil. Chemosphere. 2000;40: 7–10. 10.1016/S0045-6535(99)00216-7 10665438

[20] Binet P, Portal JM, Leyval C. Application of GC–MS to the study of anthracene disappearance in the rhizosphere of ryegrass. Org Geochem. 2001;32: 217–222. 10.1016/S0146-6380(00)00168-6

[21] Chiapusio G, Pujol S, Toussaint ML, Badot PM, Binet P. Phenanthrene toxicity and dissipation in rhizosphere of grassland plants (Lolium perenne L. and Trifolium pratense L.) in three spiked soils. Plant Soil. 2007;294: 103–112. 10.1007/s11104-007-9234-4

[22] Tejeda-Agredano MC, Gallego S, Vila J, Grifoll M, Ortega-Calvo JJ, Cantos M. Influence of the sunflower rhizosphere on the biodegradation of PAHs in soil. Soil Biol Biochem. 2013;57: 830–840. 10.1016/j.soilbio.2012.08.008

[23] Louvel B, Cébron A, Leyval C. Root exudates affect phenanthrene biodegradation, bacterial community and functional gene expression in sand microcosms. Int Biodeterior Biodegrad. 2011;65: 947–953. 10.1016/j.ibiod.2011.07.003

[24] Liste H-H, Prutz I. Plant performance, dioxygenase-expressing rhizosphere bacteria, and biodegradation of weathered hydrocarbons in contaminated soil. Chemosphere. 2006;62: 1411–1420. 10.1016/j.chemosphere.2005.05.018 15996713

[25] Hinsinger P, Gobran GR, Gregory PJ, Wenzel WW. Rhizosphere geometry and heterogeneity arising from root-mediated physical and chemical processes. New Phytol. 2005;168: 293–303. 10.1111/j.1469-8137.2005.01512.x 16219069

[26] Shann JR, Boyle J. Influence of plant species on in situ rhizosphere degradation. 1994;

[27] Marschner P, Yang C-H, Lieberei R, Crowley D. Soil and plant specific effects on bacterial community composition in the rhizosphere. Soil Biol Biochem. 2001;33: 1437–1445. 10.1016/S0038-0717(01)00052-9

[28] Anderson TA, Guthrie EA, Walton BT. Bioremediation. EST. 1993;27: 2631–2636.

[29] D’Orazio V, Ghanem A, Senesi N. Phytoremediation of pyrene contaminated soils by different plant species. CLEAN–Soil Air Water. 2013;41: 377–382.

[30] Phillips LA, Germida JJ, Farrell RE, Greer CW. Hydrocarbon degradation potential and activity of endophytic bacteria associated with prairie plants. Soil Biol Biochem. 2008;40: 3054–3064.

[31] Corgié SC, Joner EJ, Leyval C. Rhizospheric degradation of phenanthrene is a function of proximity to roots. Plant Soil. 2003;257: 143–150. 10.1023/A:1026278424871

[32] Corgié S, Beguiristain T, Leyval C. Spatial distribution of bacterial communities and phenanthrene degradation in the rhizosphere of Lolium perenne L. Appl Environ Microbiol. 2004;70: 3552–3557. 15184156 10.1128/AEM.70.6.3552-3557.2004 PMC427768

[33] Ma B, Wang J, Xu M, He Y, Wang H, Wu L, et al Evaluation of dissipation gradients of polycyclic aromatic hydrocarbons in rice rhizosphere utilizing a sequential extraction procedure. Environ Pollut. 2012;162: 413–421. 10.1016/j.envpol.2011.10.034 22243893

[34] Ling W, Dang H, Liu J. In situ gradient distribution of polycyclic aromatic hydrocarbons (PAHs) in contaminated rhizosphere soil: a field study. J Soils Sediments. 2013;13: 677–685.

[35] Hafner S, Wiesenberg GB, Stolnikova E, Merz K, Kuzyakov Y. Spatial distribution and turnover of root-derived carbon in alfalfa rhizosphere depending on top- and subsoil properties and mycorrhization. Plant Soil. 2014;380: 101–115. 10.1007/s11104-014-2059-z

[36] Demougeot-Renard H, de Fouquet C, Renard P. Forecasting the number of soil samples required to reduce remediation cost uncertainty. J Environ Qual. 2004;33: 1694–1702. 15356229 10.2134/jeq2004.1694

[37] Demougeot-Renard H, De Fouquet C. Geostatistical approach for assessing soil volumes requiring remediation: Validation using lead-polluted soils underlying a former smelting works. Environ Sci Technol. 2004;38: 5120–5126. 15506207 10.1021/es0351084

[38] De Fouquet C, Benoit Y, Carpentier C, Fricaudet B. Uncertainties on the Extension of a Polluted Zone. American Society of Mechanical Engineers; 2011 pp. 1307–1312.

[39] Faucheux C, Lefebvre E, De Fouquet C, Benoit Y, Fricaudet B, Carpentier C, et al. Characterisation of a hydrocarbon polluted soil by an intensive multi-scale sampling. 2008. pp. 961–970.

[40] De Fouquet C, Benoit Y, Carpentier C, Faucheux C, Fricaudet B. On site survey of organic pollutions: some results of the LOQUAS project. Proceedings Consoil, Salzburg; 2010.

[41] Bengtsson G, Törneman N. A spatial approach to environmental risk assessment of PAH contamination. Risk Anal. 2009;29: 48–61. 10.1111/j.1539-6924.2008.01128.x 18808392

[42] Bengtsson G, Törneman N, Yang X. Spatial uncoupling of biodegradation, soil respiration, and PAH concentration in a creosote contaminated soil. Environ Pollut. 2010;158: 2865–2871. 10.1016/j.envpol.2010.06.010 20630638

[43] Bengtsson G, Törneman N, Lipthay J, Sørensen S. Microbial Diversity and PAH Catabolic Genes Tracking Spatial Heterogeneity of PAH Concentrations. Microb Ecol. 2013;65: 91–100. 10.1007/s00248-012-0112-0 22940734

[44] Johnsen AR, Styrishave B, Aamand J. Quantification of small-scale variation in the size and composition of phenanthrene-degrader populations and PAH contaminants in traffic-impacted topsoil. FEMS Microbiol Ecol. 2014;88: 84–93. 10.1111/1574-6941.12272 24344982

[45] Törneman N, Yang X, Bååth E, Bengtsson G. Spatial covariation of microbial community composition and polycyclic aromatic hydrocarbon concentration in a creosote‐polluted soil. Environ Toxicol Chem. 2008;27: 1039–1046. 10.1897/07-440.1 18419193

[46] Mukherjee S, Juottonen H, Siivonen P, Lloret Quesada C, Tuomi P, Pulkkinen P, et al Spatial patterns of microbial diversity and activity in an aged creosote-contaminated site. ISME J. 2014;8: 2131–2142. 10.1038/ismej.2014.151 25105905 PMC4184007

[47] Ouvrard S, Barnier C, Bauda P, Beguiristain T, Biache C, Bonnard M, et al In Situ Assessment of Phytotechnologies for Multicontaminated Soil Management. Int J Phytoremediation. 2011;13: 245–263. 10.1080/15226514.2011.568546 22046763

[48] Cébron A, Faure P, Lorgeoux C, Ouvrard S, Leyval C. Experimental increase in availability of a PAH complex organic contamination from an aged contaminated soil: Consequences on biodegradation. Environ Pollut. 2013;177: 98–105. 10.1016/j.envpol.2013.01.043 23500046

[49] Reid BJ, Stokes JD, Jones KC, Semple KT. Nonexhaustive cyclodextrin-based extraction technique for the evaluation of PAH bioavailability. Environ Sci Technol. 2000;34: 3174–3179.

[50] Keith LH, Telliard WA. Priority pollutants I—a perspective view. Environ Sci Technol. 1979;13: 416–423.

[51] Cébron A, Norini M-P, Beguiristain T, Leyval C. Real-Time PCR quantification of PAH-ring hydroxylating dioxygenase (PAH-RHD_α_) genes from Gram positive and Gram negative bacteria in soil and sediment samples. J Microbiol Methods. 2008;73: 148–159. 10.1016/j.mimet.2008.01.009 18329116

[52] Thion C, Cébron A, Beguiristain T, Leyval C. Long-term in situ dynamics of the fungal communities in a multi-contaminated soil are mainly driven by plants. FEMS Microbiol Ecol. 2012;82: 169–181. 10.1111/j.1574-6941.2012.01414.x 22587649

[53] Lueders T, Wagner B, Claus P, Friedrich MW. Stable isotope probing of rRNA and DNA reveals a dynamic methylotroph community and trophic interactions with fungi and protozoa in oxic rice field soil. Environ Microbiol. 2004;6: 60–72. 10.1046/j.1462-2920.2003.00535.x 14686942

[54] Felske A, Akkermans ADL, De Vos WM. Quantification of 16S rRNAs in Complex Bacterial Communities by Multiple Competitive Reverse Transcription-PCR in Temperature Gradient Gel Electrophoresis Fingerprints. Appl Environ Microbiol. 1998;64: 4581–4587. 9797325 10.1128/aem.64.11.4581-4587.1998 PMC106687

[55] Chilès J-P, Delfiner P. Geostatistics: Modeling spatial uncertainty 2nd edition 2nd edition. Wiley; 2012.

[56] Hinsinger P, Plassard C, Tang C, Jaillard B. Origins of root-mediated pH changes in the rhizosphere and their responses to environmental constraints: A review. Plant Soil. 2003;248: 43–59. 10.1023/A:1022371130939

[57] Rousk J, Baath E, Brookes PC, Lauber CL, Lozupone C, Caporaso JG, et al Soil bacterial and fungal communities across a pH gradient in an arable soil. ISME J. 2010;4: 1340–1351. 10.1038/ismej.2010.58 20445636

[58] Walker TS, Bais HP, Grotewold E, Vivanco JM. Root exudation and rhizosphere biology. Plant Physiol. 2003;132: 44–51. 12746510 10.1104/pp.102.019661 PMC1540314

[59] Yang C-H, Crowley DE. Rhizosphere Microbial Community Structure in Relation to Root Location and Plant Iron Nutritional Status. Appl Environ Microbiol. 2000;66: 345–351. 10.1128/AEM.66.1.345-351.2000 10618246 PMC91828

[60] Baudoin E, Benizri E, Guckert A. Impact of growth stage on the bacterial community structure along maize roots, as determined by metabolic and genetic fingerprinting. Appl Soil Ecol. 2002;19: 135–145. 10.1016/S0929-1393(01)00185-8

[61] Broeckling CD, Broz AK, Bergelson J, Manter DK, Vivanco JM. Root Exudates Regulate Soil Fungal Community Composition and Diversity. Appl Environ Microbiol. 2008;74: 738–744. 18083870 10.1128/AEM.02188-07 PMC2227741

[62] Haichar F el Z, Marol C, Berge O, Rangel-Castro JI, Prosser JI, Balesdent J, et al Plant host habitat and root exudates shape soil bacterial community structure. ISME J. 2008;2: 1221–1230. 10.1038/ismej.2008.80 18754043

[63] Vos M, Wolf AB, Jennings SJ, Kowalchuk GA. Micro-scale determinants of bacterial diversity in soil. FEMS Microbiol Rev. 2013;37: 936–954. 10.1111/1574-6976.12023 23550883

[64] Becker JM, Parkin T, Nakatsu CH, Wilbur JD, Konopka A. Bacterial activity, community structure, and centimeter-scale spatial heterogeneity in contaminated soil. Microb Ecol. 2006;51: 220–231. 16463134 10.1007/s00248-005-0002-9

[65] Oliveira V, Gomes N, Almeida A, Silva A, Simões MM, Smalla K, et al Hydrocarbon contamination and plant species determine the phylogenetic and functional diversity of endophytic degrading bacteria. Mol Ecol. 2014;23: 1392–1404. 24765659 10.1111/mec.12559

[66] Siciliano SD, Germida JJ, Banks K, Greer CW. Changes in Microbial Community Composition and Function during a Polyaromatic Hydrocarbon Phytoremediation Field Trial. Appl Environ Microbiol. 2003;69: 483–489. 12514031 10.1128/AEM.69.1.483-489.2003 PMC152433

[67] Allard A-S, Remberger M, Neilson AH. The negative impact of aging on the loss of PAH components in a creosote-contaminated soil. Int Biodeterior Biodegrad. 2000;46: 43–49. 10.1016/S0964-8305(00)00050-0

[68] Cébron A, Beguiristain T, Faure P, Norini M-P, Masfaraud J-F, Leyval C. Influence of Vegetation on the In Situ Bacterial Community and Polycyclic Aromatic Hydrocarbon (PAH) Degraders in Aged PAH-Contaminated or Thermal-Desorption-Treated Soil. Appl Environ Microbiol. 2009;75: 6322–6330. 10.1128/AEM.02862-08 19633127 PMC2753067

[69] Khan S, Hesham AE-L, Qing G, Shuang L, He J. Biodegradation of pyrene and catabolic genes in contaminated soils cultivated with Lolium multiflorum L. J Soils Sediments. 2009;9: 482–491.

[70] Aprill W, Sims RC. Evaluation of the use of prairie grasses for stimulating polycyclic aromatic hydrocarbon treatment in soil. Chemosphere. 1990;20: 253–265. 10.1016/0045-6535(90)90100-8

[71] Soleimani M, Afyuni M, Hajabbasi MA, Nourbakhsh F, Sabzalian MR, Christensen JH. Phytoremediation of an aged petroleum contaminated soil using endophyte infected and non-infected grasses. Chemosphere. 2010;81: 1084–1090. 10.1016/j.chemosphere.2010.09.034 20961596

[72] Hertenberger G, Zampach P, Bachmann G. Plant species affect the concentration of free sugars and free amino acids in different types of soil. J Plant Nutr Soil Sci. 2002;165: 557–565.

