# Supplementary figures and images for: Mapping the Centimeter-Scale Spatial Variability of PAHs and Microbial Populations in the Rhizosphere of Two Plants
Source: PLoS One. 2015 Nov 23;10(11):e0142851. doi: 10.1371/journal.pone.0142851 (PMC4657893; doi:10.1371/journal.pone.0142851)

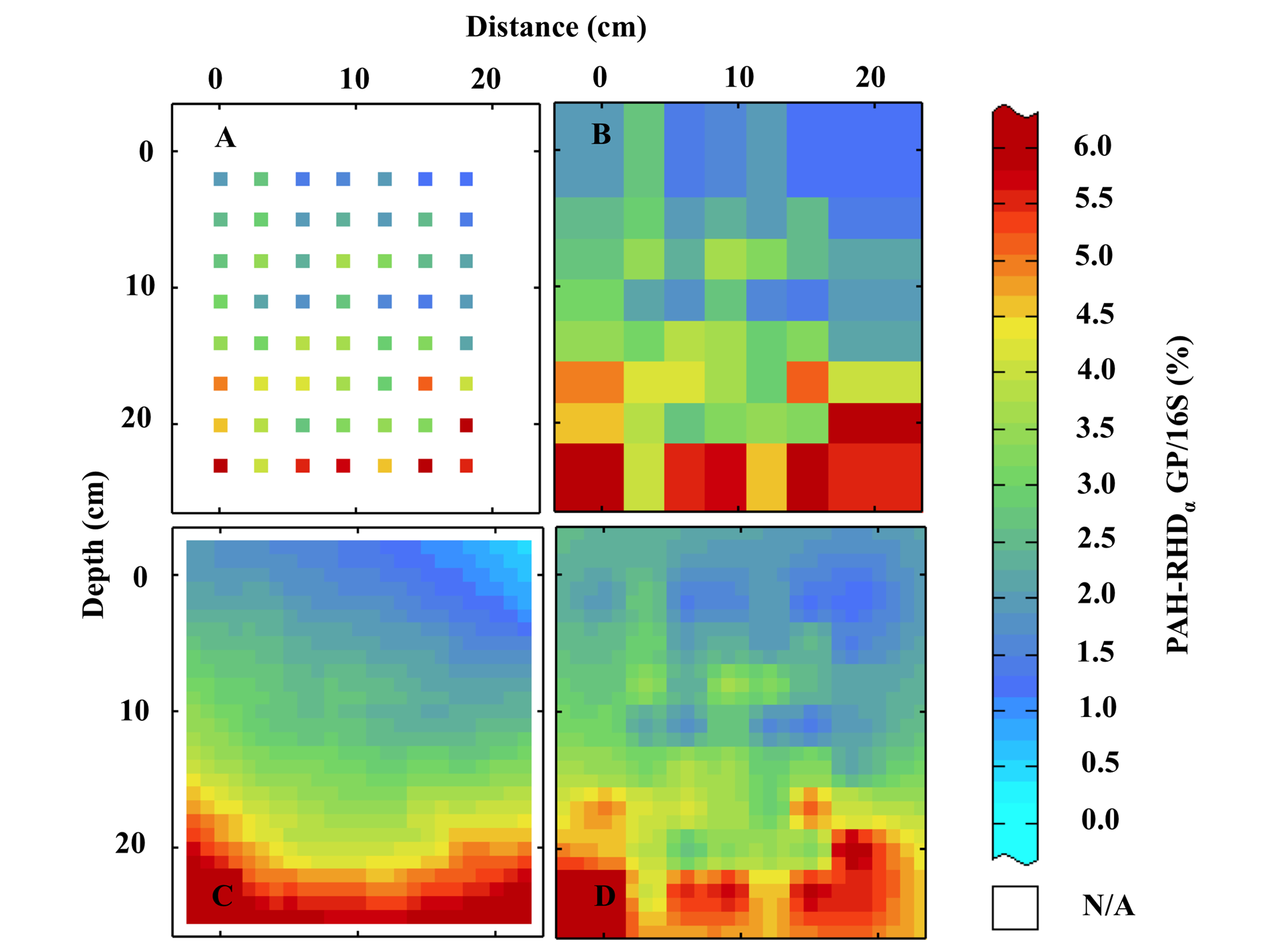

Supplement: S1 Fig — A: measured data point, B: data point estimated by nearest neighboring, C: kriging estimation, F: estimation by inverse distances, with 1/D3. N/A: non-available data. (TIF) [file pone.0142851.s001.tif]

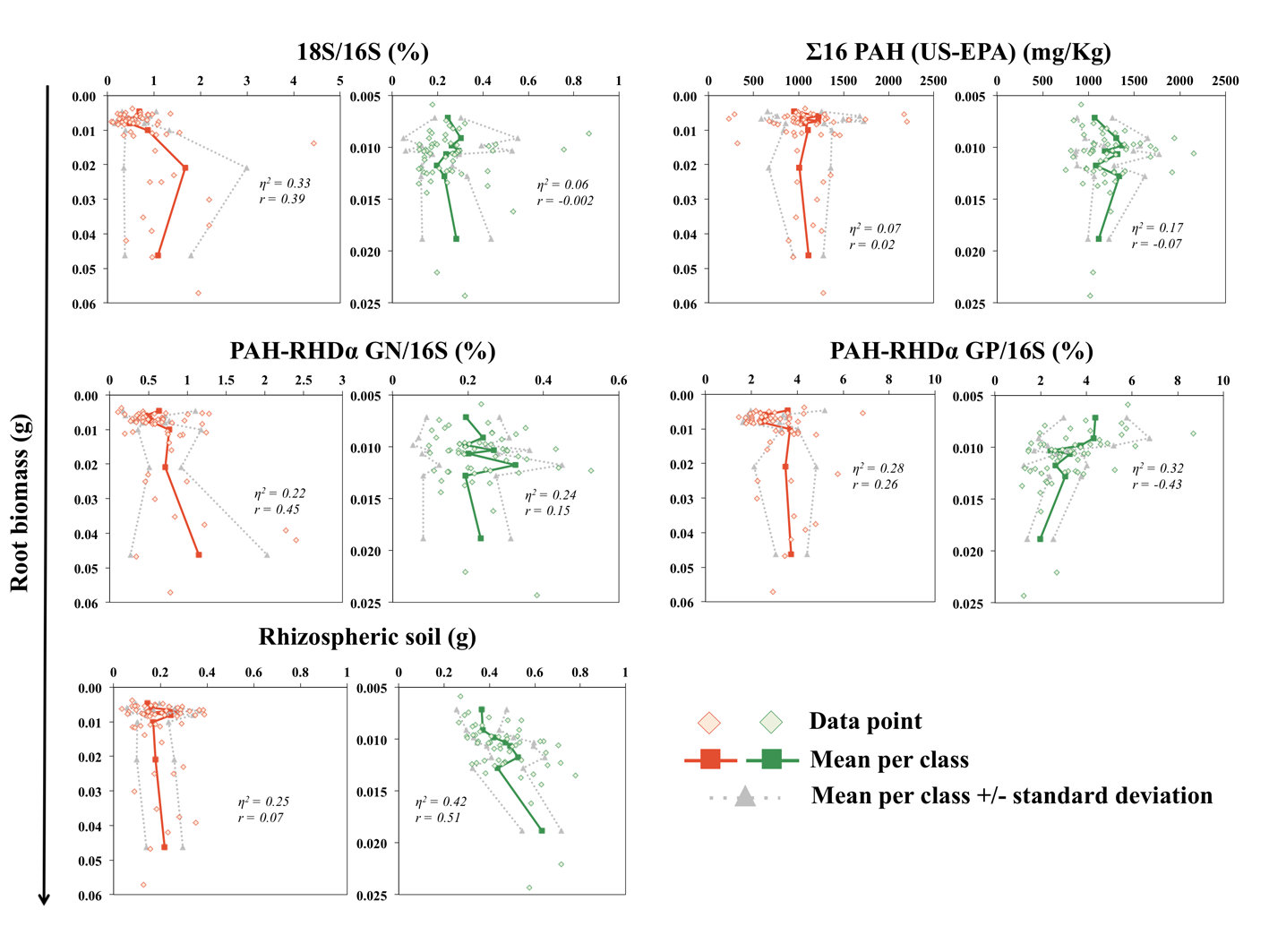

Supplement: S2 Fig — Correlation clouds show the data from the 56 soil samples (diamonds). Mean values (n = 7) per class (squares and full line) and class standard deviations (triangles and dotted line) are represented. For each relationship, the Spearman’s correlation coefficient (r) and the non-linear correlation coefficient (η2) were calculated. (TIF) [file pone.0142851.s002.tif]
