# Supplementary material for: Mapping the Centimeter-Scale Spatial Variability of PAHs and Microbial Populations in the Rhizosphere of Two Plants
Source: PLoS One. 2015 Nov 23;10(11):e0142851. doi: 10.1371/journal.pone.0142851 (PMC4657893; doi:10.1371/journal.pone.0142851)
Supplement: S1 Table — Values in bold correspond to significant correlation between variables (p<0.05). (DOCX) [file pone.0142851.s003.docx]

| Variables | Depth | %18S_alf | %18S_Rye | % PAH-RHD_α_ GN_Alf | % PAH-RHD_α_ GN_Rye | | % PAH-RHD_α_ GP_Alf | % PAH-RHD_α_ GP_Rye | Root biomass_Alf | Root biomass_Rye | Rhizo. soil_Alf | Rhizo. soil_Rye | 16 PAH_Alf | 16 PAH_Rye | 2-3 rings PAH_Alf | 2-3 rings PAH_Rye | 4 rings PAH_Alf | 4 rings PAH_Rye | 5-6 rings PAH_Alf | 5-6 rings PAH_Rye |
| --- | --- | --- | --- | --- | --- | --- | --- | --- | --- | --- | --- | --- | --- | --- | --- | --- | --- | --- | --- | --- |
| Depth | **1** | -0,215 | **-0,326** | -0,168 | | **-0,298** | 0,121 | **0,537** | **-0,636** | **-0,529** | **0,286** | **-0,517** | -0,032 | -0,057 | -0,087 | -0,139 | -0,018 | -0,058 | 0,007 | 0,001 |
| %18S_alf | -0,215 | **1** | -0,183 | **0,523** | | **-0,446** | 0,245 | -0,260 | **0,384** | 0,188 | -0,186 | 0,167 | -0,113 | 0,011 | -0,037 | 0,111 | -0,089 | -0,002 | -0,157 | -0,009 |
| %18S_Rye | **-0,326** | -0,183 | **1** | -0,194 | | **0,268** | -0,149 | -0,014 | 0,199 | 0,194 | 0,115 | **0,349** | -0,031 | 0,000 | -0,063 | 0,003 | -0,027 | 0,010 | -0,001 | -0,019 |
| % PAH-RHD_α_ GN_Alf | -0,168 | **0,523** | -0,194 | **1** | | **-0,363** | 0,150 | -0,200 | **0,344** | 0,041 | -0,122 | -0,022 | 0,020 | -0,021 | 0,026 | -0,006 | 0,002 | -0,032 | 0,025 | -0,003 |
| % PAH-RHD_α_ GN_Rye | **-0,298** | **-0,446** | **0,268** | **-0,363** | | **1** | -0,142 | 0,059 | 0,185 | 0,078 | 0,089 | 0,077 | -0,102 | 0,003 | -0,087 | 0,014 | -0,129 | -0,001 | -0,062 | -0,005 |
| % PAH-RHD_α_ GP_Alf | 0,121 | 0,245 | -0,149 | 0,150 | | -0,142 | **1** | -0,212 | 0,029 | -0,183 | 0,170 | 0,045 | -0,218 | -0,091 | -0,120 | -0,104 | -0,198 | -0,123 | -0,244 | -0,037 |
| % PAH-RHD_α_ GP_Rye | **0,537** | -0,260 | -0,014 | -0,200 | | 0,059 | -0,212 | **1** | **-0,322** | -0,245 | 0,101 | **-0,308** | -0,007 | -0,091 | -0,092 | -0,210 | 0,018 | -0,142 | 0,040 | 0,022 |
| Root biomass_Alf | **-0,636** | **0,384** | 0,199 | **0,344** | | 0,185 | 0,029 | **-0,322** | **1** | **0,323** | 0,072 | **0,464** | 0,016 | -0,073 | -0,001 | -0,040 | -0,016 | -0,090 | 0,051 | -0,063 |
| Root biomass_Rye | **-0,529** | 0,188 | 0,194 | 0,041 | | 0,078 | -0,183 | -0,245 | **0,323** | **1** | **-0,315** | **0,512** | -0,053 | -0,068 | 0,022 | 0,020 | -0,043 | -0,043 | -0,103 | -0,132 |
| Rhizo. soil_Alf | **0,286** | -0,186 | 0,115 | -0,122 | | 0,089 | 0,170 | 0,101 | 0,072 | **-0,315** | **1** | 0,007 | -0,141 | 0,124 | -0,174 | 0,100 | -0,123 | 0,120 | -0,089 | 0,132 |
| Rhizo. soil_Rye | **-0,517** | 0,167 | **0,349** | -0,022 | | 0,077 | 0,045 | **-0,308** | **0,464** | **0,512** | 0,007 | **1** | -0,024 | -0,023 | -0,021 | 0,034 | 0,003 | -0,028 | -0,040 | -0,052 |
| 16 PAH_Alf | -0,032 | -0,113 | -0,031 | 0,020 | | -0,102 | -0,218 | -0,007 | 0,016 | -0,053 | -0,141 | -0,024 | **1** | 0,114 | **0,760** | 0,012 | **0,928** | 0,131 | **0,945** | 0,131 |
| 16 PAH_Rye | -0,057 | 0,011 | 0,000 | -0,021 | | 0,003 | -0,091 | -0,091 | -0,073 | -0,068 | 0,124 | -0,023 | 0,114 | **1** | -0,032 | **0,917** | 0,196 | **0,976** | 0,125 | **0,951** |
| 2-3 rings PAH_Alf | -0,087 | -0,037 | -0,063 | 0,026 | | -0,087 | -0,120 | -0,092 | -0,001 | 0,022 | -0,174 | -0,021 | **0,760** | -0,032 | **1** | -0,083 | **0,520** | -0,012 | **0,548** | -0,032 |
| 2-3 rings PAH_Rye | -0,139 | 0,111 | 0,003 | -0,006 | | 0,014 | -0,104 | -0,210 | -0,040 | 0,020 | 0,100 | 0,034 | 0,012 | **0,917** | -0,083 | **1** | 0,119 | **0,913** | -0,007 | **0,779** |
| 4 rings PAH_Alf | -0,018 | -0,089 | -0,027 | 0,002 | | -0,129 | -0,198 | 0,018 | -0,016 | -0,043 | -0,123 | 0,003 | **0,928** | 0,196 | **0,520** | 0,119 | **1** | 0,198 | **0,904** | 0,208 |
| 4 rings PAH_Rye | -0,058 | -0,002 | 0,010 | -0,032 | | -0,001 | -0,123 | -0,142 | -0,090 | -0,043 | 0,120 | -0,028 | 0,131 | **0,976** | -0,012 | **0,913** | 0,198 | **1** | 0,146 | **0,880** |
| 5-6 rings PAH_Alf | 0,007 | -0,157 | -0,001 | 0,025 | | -0,062 | -0,244 | 0,040 | 0,051 | -0,103 | -0,089 | -0,040 | **0,945** | 0,125 | **0,548** | -0,007 | **0,904** | 0,146 | **1** | 0,154 |
| 5-6 rings PAH_Rye | 0,001 | -0,009 | -0,019 | -0,003 | | -0,005 | -0,037 | 0,022 | -0,063 | -0,132 | 0,132 | -0,052 | 0,131 | **0,951** | -0,032 | **0,779** | 0,208 | **0,880** | 0,154 | **1** |
